# Clinico-Epidemiological Profile of Patients Presenting With Acute Chest Discomfort in Emergency Medicine Department of a Tertiary Care Hospital in Uttarakhand, India: A Prospective Observational Study

**DOI:** 10.7759/cureus.44681

**Published:** 2023-09-04

**Authors:** Anmol Chanda, Nidhi Kaeley, Barun Kumar, Meenakshi Khapre

**Affiliations:** 1 Emergency Medicine, All India Institute of Medical Sciences, Rishikesh, Rishikesh, IND; 2 Cardiology, All India Institute of Medical Sciences, Rishikesh, Rishikesh, IND; 3 Community and Family Medicine, All India Institute of Medical Sciences, Rishikesh, Rishikesh, IND

**Keywords:** point of care ecg, mortality indicators, premature acute coronary syndrome, acute chest discomfort, clinico-epidemilogical profile

## Abstract

Background

Acute chest discomfort is a common presenting complaint in the emergency department. There is a paucity of studies related to clinico-epidemiological profile of patients with acute chest discomfort in the emergency department (ED). Hence, we intended to conduct the study to address the dearth of research in this field.

Aims and objectives

The primary objective of this study was to study the clinico-epidemiological profile of patients with acute chest discomfort presenting to the ED. The secondary objectives were to assess the prevalence of premature acute coronary syndrome (ACS), to study the ED disposition and final hospital discharge diagnosis, and to assess the predictors of 24-hour mortality in such patients.

Methods

A prospective observational study of patients presenting with acute chest discomfort was conducted in the emergency medicine department of a tertiary care hospital. We included adults above the age of 18 years from December 2021 to December 2022 and excluded trauma patients. A standardized form was used to document patient demographic patterns, comorbidities, chest discomfort description, physical findings, investigations, consultations, ED management, and disposition. Variables having p-value ≤ 0.05 were considered to be significant.

Results

A total of 200 patients were included. The most common cause of chest discomfort in the ED was cardiac, accounting for 48.5% (n = 97) of patients. The most common cardiac cause of acute chest discomfort was ST-elevation myocardial infarction (STEMI) ~ 21% (n = 42). Cardiac diagnosis was associated with the maximum number of admissions (≈80%; n = 78). The prevalence of premature ACS was 13.9% (n = 10). A 24-hour mortality was significantly associated with male gender, ambulance transport, history of coronary artery disease, and hypoxia and hypotension at the initial presentation.

Conclusions

ACS followed by respiratory causes are the predominant etiologies of acute chest discomfort in the ED. Knowledge of the differential diagnosis of acute chest discomfort in the ED can aid in prompt diagnosis and delivery of lifesaving treatment to these patients.

## Introduction

Acute chest discomfort is described as the recent onset of pain, pressure, or tightness in the anterior thorax between the xiphoid, suprasternal notch, and both mid-axillary lines [1]. Discomfort can be categorized as somatic or visceral in origin. Somatic discomfort includes pain from the musculoskeletal structures, coverings of major organs, and dermal tissues, whereas visceral discomfort includes pain from organs like the heart, liver, etc. [2].

Differentiating between life-threatening and non-life-threatening causes of chest pain is a key aspect of emergency medicine [3].

Potentially life-threatening causes include acute coronary syndrome (ACS), aortic dissection, pulmonary embolism (PE), ruptured aortic aneurysm, and tension pneumothorax. In patients with non-life-threatening chest discomfort, a diagnosis can be made only after a thorough work-up [2].

Studies have reported that patients with non-cardiac chest discomfort and a normal coronary angiogram may be suffering from various psychiatric disorders, most commonly panic disorder, anxiety, and depression [2].

ACS is a group of conditions caused by a sudden blockage of the blood supply to the heart. It constitutes an important problem because of the devastating effect of this disease on the more active lifestyle of young adults [4].

There are no universal definitions/criteria to describe the cut-off age for premature ACS, so we defined a case with premature ACS as one below the age of 40 years as described in a study by Noeman et al. [5].

Acute chest discomfort is one of the common emergencies. There is a paucity of studies in the Indian population describing the causes, prevalence, and disposition of patients with acute chest discomfort presenting to the emergency medicine department of a tertiary care hospital. Expanding our knowledge base on the etiologies, required investigations, and management of patients with acute chest discomfort will aid emergency physicians in focused, appropriate, and cost-effective patient care. It can aid in the formulation of a diagnostic algorithm and protocolized management of acute chest discomfort.

## Materials and methods

A prospective observational study comprising 200 emergency department (ED) patients with acute chest discomfort was conducted in the ED of All India Institute of Medical Sciences, Rishikesh. It is a tertiary care government hospital situated in the state of Uttarakhand, India. The study population consisted of patients above the age of 18 years presenting to ED with acute non-traumatic chest discomfort from December 2021 to December 2022. We took 200 as the sample size following the convenient consecutive sampling method.

The aims of the study were to study the clinico-epidemiological profile of patients presenting with acute chest discomfort to the ED, to assess the prevalence of premature ACS, to study the ED disposition and final hospital diagnosis or discharge, to assess the predictors of mortality in patients with acute chest discomfort.

The attending emergency medicine resident used a standardized form to document patient demographics, comorbidities, chest discomfort description, physical findings, investigations, consultations, ED management, and disposition. The included patients were followed up in the hospital till discharge/death for final diagnosis and outcome.

All data was entered into an Excel sheet (Microsoft Corporation, Redmond, Washington) and analyzed using the SPSS software version 23.0 (IBM Corp., Armonk, NY). Patient demographic and clinical characteristics and the outcome were measured as mean and standard deviation (SD) for continuous variables and as frequencies and percentages for categorical variables.

Data presentation was done graphically wherever appropriate using histograms for quantitative data and bar charts/pie charts for qualitative data. The prevalence of ACS was expressed in proportion.

The association between two categorical variables was explored using the Chi-square test. In case the expected frequency in the contingency tables was found to be less than five for >25% of the cells, Fisher’s exact test was used instead.

Associations between variables where one is continuous and one is categorical were explored using independent sample “t”-test when the categorical variable had two categories and one-way ANOVA when it had more than two categories. If data was found to be non-normally distributed, appropriate non-parametric tests in the form of the Wilcoxon Mann-Whitney U Test/Kruskal-Wallis Test were used for these comparisons. Statistical significance was kept at p < 0.05.

## Results

A total of 200 patients with acute chest discomfort who presented to the ED between December 2021 and December 2022 were included in our study. Demographic characteristics of these patients are presented in Table 1.

**Table 1:** Demographic characteristics of all the patients with acute chest discomfort (n = 200)

Basic details	Mean ± SD; n (%)
Age (years)	53.26 ± 16.23
*Age*	
<40 years	47 (23.5%)
>40 years	153 (76.5%)
*Gender*	
Male	124 (62.0%)
Female	76 (38.0%)
*State*	
Uttar Pradesh	95 (47.5%)
Uttarakhand	96 (48.0%)
West Bengal	1 (0.5%)
Maharashtra	2 (1.0%)
Delhi	2 (1.0%)
Others	4 (2.0%)
*Mode of prehospital transport*	
Public transport	2 (1.0%)
Private vehicle	126 (64.9%)
Ambulance	66 (34.0%)

In our study, the mean ± SD of age (Years) was 53.26 ± 16.23 (Table 1); 23.5% (n = 47) of patients were ≤ 40 years of age. Clearly, males (62%; n = 124) dominated females (38%; n = 76) as shown in Table 1. Around 60% (n = 126) of patients were transported by private vehicles followed by ambulance (34%; n = 66) (Table 1).

About 46.5% (n = 93) of patients presented with chest pain and described it as pressure-like and radiating to bilateral arms as shown in Table 2. The mean (± SD) heart rate (BPM) of the patients was 91.62 ± 23.59. The mean (± SD) systolic BP (mmHg) of the patients was 112.61 ± 20.64. The mean (± SD) diastolic BP (mmHg) of the patients was 72.65 ± 11.99 (Table 2).

**Table 2:** Clinical characteristics of all the patients with acute chest discomfort (n = 200) COPD: Chronic obstructive pulmonary disease; HEART: History, electrocardiogram, age, risk factors, and troponin.

Vitals	Mean ± SD
Heart rate (BPM)	91.62 ± 23.59
Systolic BP (mmHg)	112.61 ± 20.64
Diastolic BP (mmHg)	72.65 ± 11.99
*Time since symptom onset*	*n (%)*
Hours	86 (43.0%)
Days	98 (49.0%)
Months	13 (6.5%)
Weeks	3 (1.5%)
*HEART score*	
0 to 3	44 (81.5%)
≥4	10 (18.5%)
*Chest pain description*	
Burning type	33 (16.5%)
Pleuritic	57 (28.5%)
Pressure like, radiating	93 (46.5%)
Sharp non-radiating	10 (5.0%)
Others	7 (3.5%)
*Comorbidities*	*n (%)*
Smoker	97 (48.5%)
Hypertension	71 (35.5%)
Diabetes	61 (30.5%)
None	51 (25.5%)
Coronary artery disease	39 (19.5%)
COPD	22 (11.0%)
Congestive heart failure	11 (5.5%)
Chronic kidney disease	11 (5.5%)
Pulmonary TB	9 (4.5%)
Chronic alcohol intake	7 (3.5%)
Immunocompromised state	5 (2.5%)
Malignancy	4 (2.0%)

The majority of patients presenting with chest pain to the ED were smokers (48.5%; n = 97) as shown in Table 2. The most common comorbidities among the patients were hypertension (35.5%; n = 71) and diabetes mellitus (30.5%; n = 61) as shown in Table 2.

About 32.0% (n = 64) of the patients had normal sinus rhythm in the ECG on admission; 25.0% (n = 50) of the patients had ST-elevation myocardial infarction (STEMI) in the ECG on admission, and 12.5% (n = 25) of the patients had non-ST-elevation acute coronary syndrome (NSTE-ACS) in the ECG on admission as shown in Table 3.

**Table 3:** Point-of-care investigations done in ED STEMI: ST-elevation myocardial infarction; NSTE-ACS: Non-ST-elevation acute coronary syndrome; LVEF: Left ventricular ejection fraction; LRTI: Lower respiratory tract infection; CXR: chest X-ray; POCUS: Point-of-care ultrasound.

ECG on admission	n (%)
Normal sinus rhythm	64 (32%)
STEMI	50 (25%)
NSTE-ACS	25 (12.5%)
Tachycardia	44 (22%)
Bradycardia	5 (2.5%)
Others	12 (6%)
Presentation-contact interval	
<5 minutes	175 (87.5%)
5-10 minutes	24 (12.0%)
>10 minutes	1 (0.5%)
Presentation-ECG interval	
<5 minutes	18 (9.0%)
5-10 minutes	182 (91.0%)
>10 minutes	0 (0.0%)
Time for troponin I results	
<5 minutes	0 (0.0%)
5-10 minutes	6 (3.0%)
15-20 minutes	97.0%)
POCUS findings	n (%)
A profile	161 (80.5%)
B profile	12 (6.0%)
C profile	12 (6.0%)
LVEF reduced	11 (5.5%)
LVEF adequate	5 (2.5%)
Pleural effusion	1 (0.5%)
Pneumothorax	1(0.5%)
CXR findings	n (%)
Normal	142 (71.0%)
LRTI	34 (17.0%)
Pulmonary edema	17 (8.5%)
Pleural effusion	5 (2.5%)
Pneumothorax	2 (1.0%)
Lung mass	1 (0.5%)

The majority (87.5%; n = 175) of patients were attended to within five minutes of arrival by the ED physician. ECG for most patients (91%; n = 182) was done within an interval of 5-10 minutes of the ED presentation. Troponin I results were received within 15-20 minutes from the time of presentation for around 97% (n = 194) of patients (Table 3).

The majority (80.5%; n = 161) of the patients with chest pain had an "A - profile" on point-of-care ultrasound (POCUS) findings. Around two-thirds (71%; n = 142) of patients had a normal initial chest X-ray as shown in Table 3.

Five (2.5%) of the patients were thrombolyzed in the ED. All of them had a diagnosis of STEMI and were transported by ambulance. About 80% (n = 4) of the thrombolyzed participants were > 40 years of age and males. All these patients were admitted to the cardiology ward, and a facilitated percutaneous coronary intervention (PCI) was done for them. Out of those participants who underwent PCI, 82.9% (n = 29) of the patients had primary PCI, whereas 17.1% (n = 6) of the patients had facilitated PCI. Eight (4.0%) of the patients had mortality within 24 hours of admission (Table 4).

**Table 4:** Management and outcome in the emergency department ED: Emergency department; PCI: Percutaneous coronary intervention.

Management and outcome	n (%)
Thrombolysis in ED	5 (2.5%)
*ED disposition*	
Absconding from ED	1 (0.5%)
Admitted in ICU	12 (6%)
Admitted to the ward	99 (49.7%)
Died in ED	8 (4.0%)
Discharged from ED	80 (40.2%)
*Place of admission*	
Cardiology ward	73 (36.5%)
ICU	12 (6%)
Medicine ward	9 (4.5%)
Pulmonary ward	17 (8.5%)
Not admitted	89 (44.5%)
*ED length of stay (hours)*	
0-12 hours	161 (80.5%)
12-24 hours	25 (12.5%)
>24 hours	14 (7.0%)
*Type of PCI*	
Primary	29 (82.9%)
Facilitated	6 (17.1%)
24-hour mortality	8 (4.0%)

**Figure 1:**
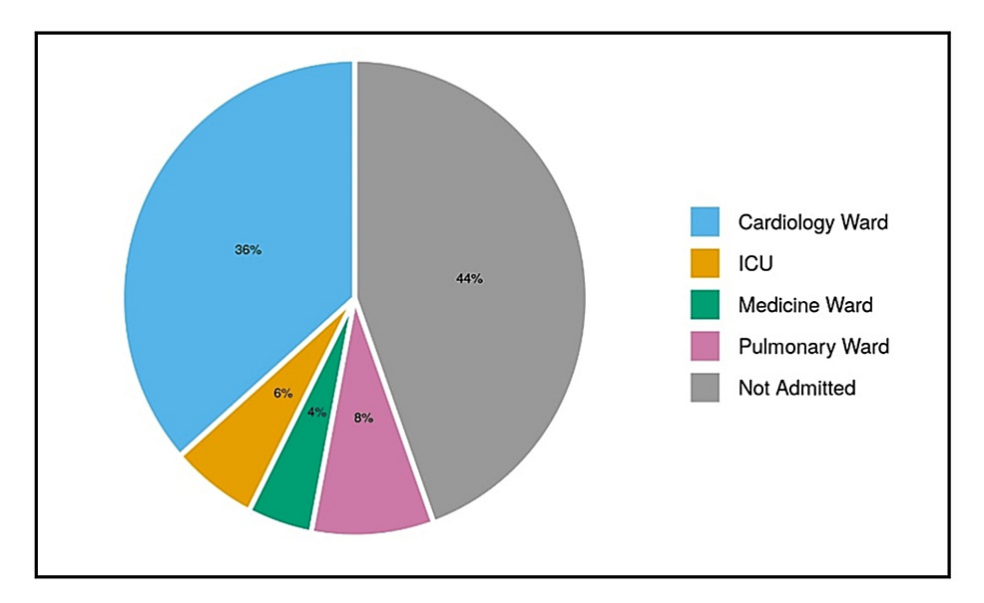
Pie chart showing the distribution of ED disposition ED: Emergency department.

About 6% (n = 12) of the patients were admitted to intensive care unit (ICU). About 49.7% (n = 99) of the patients were admitted to the ward; 4.0% (n = 8) of the patients died in ED, and 40% (n = 80) of the patients were discharged from ED as shown in Table 4 and Figure 1.

The prevalence of premature ACS that is present in <40 years old was 13.9%; 10 out of the total 72 participants had premature ACS (≤40 years of age) (Table 5).

**Table 5:** Prevalence of premature ACS ACS: Acute coronary syndrome.

Age	ACS
	n (%)
<40 years	10 (13.9%)
>40 years	62 (86.1%)
Total	72 (100.0%)

Clinico-epidemiological factors that were significantly associated (p < 0.05) with 24-hour mortality were male gender, ambulance mode of transport, comorbidities such as coronary artery disease, and congestive heart failure; threatened airway, hypoxia, and hypotension were present in most of the participants who died within 24 hours (Table 6).

**Table 6:** Predictors of 24-hour mortality in patients presenting with acute chest discomfort ***Significant at p < 0.05. ^1^Fisher's exact test.

Parameters	24-hour mortality	p-value
	(n = 8) Mean ± SD; n (%)	
Age (Years)	62.25 ± 15.23	
Gender***		0.025^1^
Male	8 (100.0%)	
Mode of prehospital transport***		0.002^1^
Ambulance	7 (100.0%)	
*Comorbidities*		
Coronary artery disease***	5 (62.5%)	0.008^1^
Congestive heart failure***	3 (37.5%)	0.006^1^
Airway: Drooling of saliva ***	4 (50.0%)	<0.001^1^
Airway: Vomitus in mouth ***	1 (12.5%)	0.040^1^
Breathing assessment: Hypoxia ***	6 (75.0%)	<0.001^1^
Circulation assessment***		<0.001^1^
Hypotension	6 (75.0%)	

**Figure 2:**
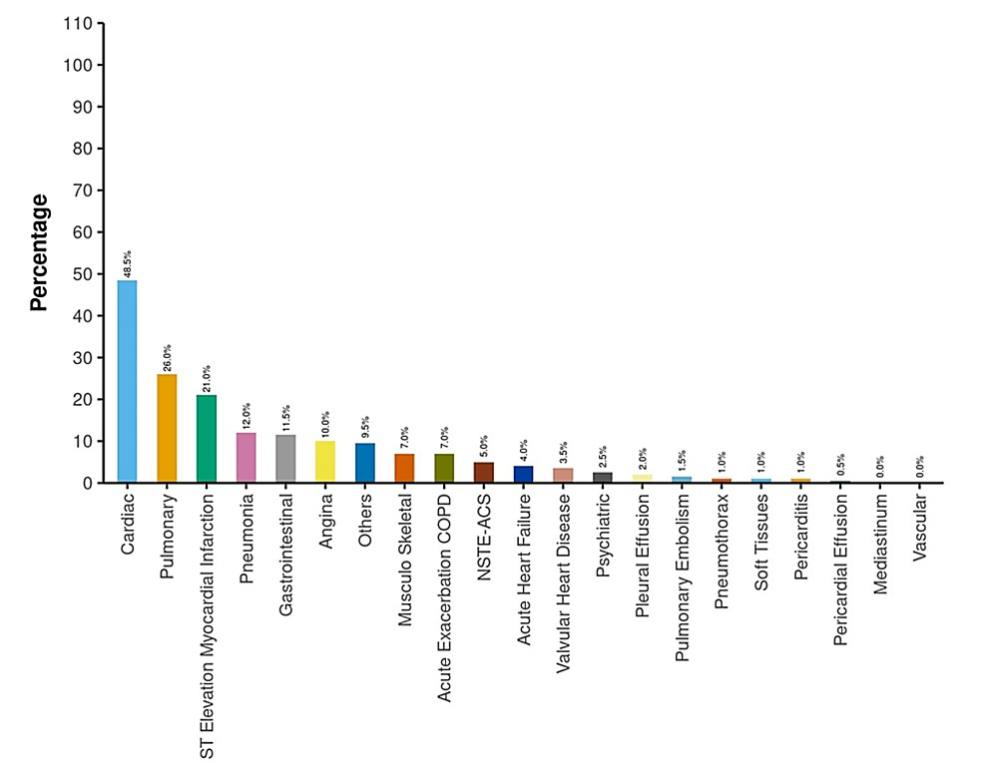
Diagnosis at the time of ED disposition NSTE-ACS: Non-ST-elevation acute coronary syndrome; ED: Emergency department; COPD: Chronic obstructive pulmonary disease.

**Figure 3:**
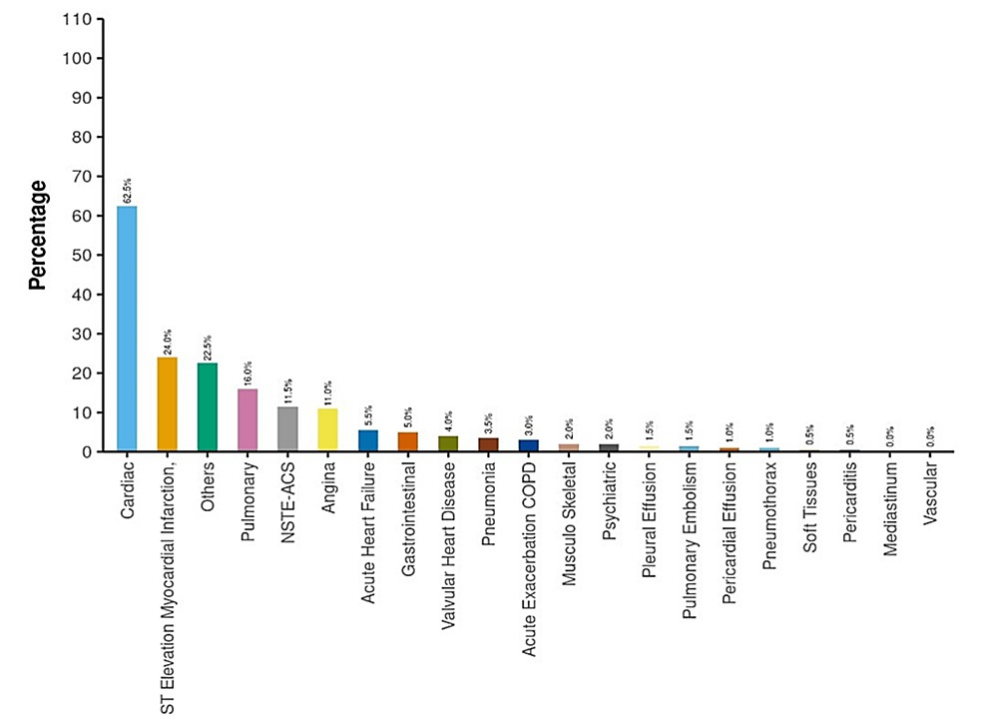
Final hospital diagnosis at the time of hospital discharge NSTE-ACS: Non-ST-elevation acute coronary syndrome; ED: Emergency department; COPD: Chronic obstructive pulmonary disease.

The most common etiology of chest discomfort in the ED and final hospital diagnosis at the time of discharge was cardiac, 48.5% (n = 97) and 62.5% (n = 125), respectively (Figures 2, 3).

The most common cardiac etiology of acute chest discomfort was STEMI ~ 21% (n = 42) (Figure 2).

## Discussion

This was a prospective observational study to evaluate the patients with non-traumatic acute chest discomfort in the tertiary care hospital of Uttarakhand.

Two hundred patients were enrolled in the study. A thorough work-up was done by the primary attending physician. The patients were then treated, and their disposition was done accordingly.

Etiology of acute chest discomfort

Participants with cardiac cause of chest pain were older than the average age with a mean (± SD) age of 56.6 years ± 14.83. Comorbidities of hypertension (47.4%; n = 46) and diabetes mellitus (42.3%; n = 41) were the most prominent in the cardiovascular disease group. These findings were similar to the study done by Geyser et al. [2]. This could be explained by the fact that non-communicable diseases are common in the geriatric population. In particular, cardiovascular patients were transported more by ambulance than by their own or private transport; this could be because of the critical illness of the cardiac patients, and a majority of patients were referred from primary care centers via ambulance.

The most common diagnosis for pulmonary cause of chest pain made in ED was pneumonia (46.2%; n = 24), which was followed by an acute exacerbation of COPD (26.9%; n = 14) followed by pleural effusion (7.7%; n = 4) and PE (5.8%; n = 3). About 13.5% (n = 7) of these patients were diagnosed with pulmonary tuberculosis at some point in their lifetime. About 75% (n = 39) of these patients were smokers, and 36.5% (n = 19) of patients were diagnosed cases of COPD, which explains the high prevalence of acute exacerbations of COPD in our spectrum of pulmonary diagnosis.

Chest pain is commonly present in 60% of patients with PE, and PE is classically said to be one of the most frequently under-reported diagnoses [6]. Therefore, a detailed history and thorough clinical examination should be done along with point-of-care investigations to rule out PE as the diagnosis for acute chest discomfort.

The prevalence of gastrointestinal causes of acute chest discomfort in our patients was 11.5% (n = 23). Gastro-esophageal reflux disease is often reported in around one-third of cases of recurrent non-cardiac chest pain [7].

Musculoskeletal cause of acute chest discomfort as final ED diagnosis was present in 7% (n = 14) of total patients. This might be a good reflection of the real frequency of musculoskeletal pathology in ED patients presenting with acute chest discomfort.

In our study, only 2.5% (n = 5) of cases were found to have somatization/psychiatric disorders. This could be attributed to the fact that detailed psychiatric evaluation is seldom done in ED patients. These patients are often referred to OPDs.

Psychiatric diseases are one of the causes of hyperventilation in patients presenting to the ED [8]; however, we should rule out the more life-threatening causes of acute chest discomfort and hyperventilation first before considering a psychiatric cause of underlying illness in these patients.

Epidemiological profile of patients with acute chest discomfort

Age and Gender

The mean age of our patients was 53.26 ± 16.23 years. About 23.5% (n = 47) of patients were ≤ 40 years of age. Clearly, males outnumbered females. These results were comparable to the previous studies done by Knockaert et al. [9] and Buntinx et al. [10]. The mean age reported by these studies was 60 years. Most of the patients reported by previous studies were more than 50 years of age. It is a known fact that non-communicable diseases such as ACS, hypertension, respiratory diseases, etc. are common among the geriatric population [11-14]. Although our population had younger patients (13.9%; n = 10) as well who presented with premature ACS, there has been an increase in cases of coronary artery disease among the younger population in the last three decades [15]. Patients with premature ACS have a similar risk factor profile, with smoking being the most common [16]. The incidence of coronary artery disease among younger patients varies between 12% and 16%, and around one-fourth of them are reported to be under 40 years of age [17].

Transportation

Around one-third of our patients were transported by ambulance. These results concurred with previous studies done by Knockaert et al. [9] and Geyser et al. [2]. The findings of the study done by Paichadze et al. are contrary to our study findings as most of the patients reached their EDs using other means of transport (91.9%; n = 18,150) than ambulance service (2.9%; n = 585) [18]. This could be because of a lack of well-developed and well-connected prehospital services in developing countries and growing illiteracy and unaffordability for these services in developing countries.

Comorbidities

The majority of participants presenting with chest discomfort to the ED were smokers (48.5%; n = 97). Smoking is a known risk factor for cardiac cause of chest discomfort. The underlying pathophysiology is that smoking causes a pro-thrombotic state, and a simultaneous reduction in the coronary reserve flow [19].

The most common comorbidities among the participants were hypertension (35.5%; n = 71) and diabetes mellitus (30.5%; n = 61). Geyser et al. [2] reported hypertension and diabetes mellitus as the most prevalent comorbidities in the cardiovascular disease group. Hypertension has been reported in around 31%-59% of patients with ACS [20,21]. Uncontrolled hypertension results in the formation of fatty deposits in the arteries and the development of vulnerable plaques whose instability or rupture is responsible for the development of ACS [22]. Diabetes mellitus is reported in approximately 30% of patients with ACS [23,24]. The underlying pathophysiology is endothelial dysfunction, plaque alteration, platelet activation, and coagulation disturbances. The interplay of these factors leads to an intense pro-thrombotic, pro-inflammatory state [25].

Point-of-care investigations: ECG/troponin I

ECG is an essential tool for critical decision-making; ECG done within ≤ 10 minutes of patient presentation to the ED is considered one of the quality indicators of care in chest discomfort management in the ED [18].

We did an ECG and point-of-care troponin I for every patient who presented with acute chest discomfort in our ED. Since we reported 36% (n = 72) of patients with acute chest discomfort as having ACS, it reinstates the fact that we should screen all the patients with acute chest discomfort with ECG and cardiac biomarkers in the ED.

In our study, the time from the patient presentation to the ED and first ED physician contact for most patients (87.5%) was less than five minutes. ECG for most of the patients (91%) was done within 5-10 minutes of the ED presentation.

In our study, the prevalence of premature ACS that is present in <40 years old is 13.9%; 10 out of the total 72 participants had premature ACS (≤40 years of age).

The last two to three decades have seen an exponential rise in coronary artery disease (CAD) cases in the young, but the data regarding premature coronary heart disease and ACS (≤ 40 years) is limited [15]. This compelled us to find the prevalence of such patients.

Emergency department disposition

Patients with pulmonary cause of acute chest discomfort were more likely to be admitted (53%; n = 27) than discharged (43.1%; n = 22). Similar results were shown by the study done by Geyser et al. [2]. One of the reasons for higher admission rates could be the need for oxygen support, non-invasive or invasive ventilation required by such patients, and also the need for intravenous antibiotics as part of their treatment.

The majority of our patients (95.7%; n = 22) with gastrointestinal causes of acute chest discomfort were discharged from the ED. Our results regarding the disposition of such patients were similar to the study done by Geyser et al. [2]. One of the reasons for the majority of patients being discharged could be the rapid onset of their symptom relief with the administration of antacids in the ED. These patients are often referred to outpatient departments for further work-up.

All of our patients presenting with musculoskeletal causes of acute chest discomfort were discharged from the ED. All patients with a psychiatric cause of acute chest discomfort were discharged from the ED after initial evaluation. Our results were similar to the findings reported by Geyser et al. [2].

Predictors of mortality in patients with acute chest discomfort

The mean (± SD) age of the patients who died within 24 hours of ED arrival was 62.25 ± 15.23 years. All of them were males. Three-fourths of the patients who died within 24 hours had a cardiac cause of acute chest discomfort. All of them were transported by ambulance to the hospital as they were critically ill.

Study limitations

We get multiple patients with multi-organ involvement. Thus, we could not attribute the symptom of acute chest discomfort to a particular system in a few of the patients.

Both geriatric and younger populations were included in the study. The etiology of acute chest discomfort can vary in different age groups. Thus, mortality parameters and risk factors of acute chest discomfort also varied in younger and geriatric populations.

Although ACS was ruled out systematically using ECG, cardiac biomarkers were followed by bedside echocardiography. However, other causes such as gastrointestinal could not be ruled out using gold standard techniques. Most of such patients were empirically diagnosed using history and clinical examination.

There was a lack of follow-up of patients who were discharged from the ED after ruling out ACS.

## Conclusions

ACS followed by respiratory diseases is the predominant cause of acute chest discomfort in the ED. The 24-hour mortality predictors of patients with acute chest discomfort include male gender, diagnosis of cardiopulmonary causes of acute chest discomfort, diagnosis of acute heart failure, transport by ambulance, comorbidities such as coronary artery disease, congestive heart failure, threatened airway, hypoxia, and hypotension on presentation. The non-cardiac causes of acute chest discomfort include respiratory, gastrointestinal, musculoskeletal, psychiatric, and miscellaneous (soft tissues, vascular, etc.). Knowledge of the differential diagnosis of acute chest discomfort in the ED can aid in prompt diagnosis and delivery of necessary treatment for these patients.
